# Biofilm formation and adhesion to bovine udder epithelium of potentially probiotic lactic acid bacteria

**DOI:** 10.3934/microbiol.2018.2.209

**Published:** 2018-03-19

**Authors:** Jonathan K. Wallis, Volker Krömker, Jan-Hendrik Paduch

**Affiliations:** Faculty II, Department for Bioprocess Engineering and Microbiology of the University of Applied Sciences and Arts Hannover, Lower Saxony, Germany

**Keywords:** probiotic potential, biofilm, adhesion, lactic acid bacteria, mastitis

## Abstract

Mastitis is one of the most important diseases threatening modern dairy herds. The idea of fighting the disease through colonising the udder with lactic acid bacteria (LAB), thereby building a beneficial biofilm, is the base for a probiotic approach towards mastitis control. The purpose of this study was to screen 13 LAB strains (eleven wild strains, two ATCC strains) inhibitory to the growth of mastitis-causing pathogens for their *in vitro* ability to form a biofilm and to adhere to bovine glandular mammary epithelium in order to assess their probiotic potential. Furthermore, we aimed to gain knowledge about the chemical nature of the adhesins involved by subjecting the bacteria to various chemical and enzymatical pre-treatments. The biofilms were grown on hydrophilic glass and on hydrophobic polypropylene in de Man, Rogosa and Sharpe (MRS) broth and afterwards quantified with a crystal violet assay. Biofilm formation was observed in all strains. However, the extent strongly depended on the strain, surface charge and medium. The adhesion assay also revealed a strong strain dependency, but this trait was also present in all of the investigated LAB isolates. Depending on the strain, chemical or enzymatical pre-treatment revealed carbohydrate molecules as well as proteins and lipids to be crucial for the adhesion of LAB to epithelial cells. The seven strains showing the strongest biofilm formation and/or adhesion represent promising candidates for further investigation in order to develop a probiotic remedy for the treatment of mastitis. Still, their safety for consumers and patients as well as their capability to colonise the udder remain to be investigated in *in vivo* studies.

## 1. Introduction

Bovine mastitis is among the most detrimental diseases the dairy industry is facing [1]. It causes considerable economic losses due to treatment costs, diagnosis and veterinary services, loss of milk caused by withdrawal time after antibiotic therapy as well as decreased milk yield and through negative effects on reproduction [2]. Recent studies have revealed that the disease can be accompanied by biofilm formation of the causative agent [3]. Other authors found an increase in biofilm formation induced by milk components possibly affecting the development of the infection [4]. Donlan [5] defines a biofilm as an assemblage of microorganisms that is irreversibly attached to a surface and enclosed in a matrix. Numerous studies associate biofilms with persistent infections and failure of antibiotic therapy, as they can offer protection from the host's immune system [6] and reduce the effect of applied antibiotics [7],[8]. Schönborn and Krömker [3] have shown that *Staphylococcus aureus* may form biofilms in infected udders. *In vitro* studies suggest that there are many more pathogens, e.g. *Eschericha coli*, *Streptococcus uberis*, *Streptococcus dysgalactiae* and coagulase negative staphylococci, possessing the ability to cause biofilm-related mastitis since they form biofilms *in vitro* [9]. In accordance with the previously mentioned findings by Anderl et al. [7] and Zahller and Stewart [8], these findings of Schönborn et al. [9] emphasise the urgent need for new approaches for treating mastitis. In addition to these studies, a great deal of effort is being put into investigating probiotic microorganisms suitable for treating or preventing udder infections [10]–[12]. The Food and Agriculture Organization of the United Nations (FAO) and the World Health Organization (WHO) define probiotics as “live microorganisms that, when administered in adequate amounts, confer a health benefit on the host” [13]. The group of lactic acid bacteria is often associated with probiotics since they have traditionally been used as starter cultures in the food industry [14] and therefore many of them are regarded as harmless to consumers. This is also expressed by the GRAS (generally recognized as safe) status given to a large number of LAB by the FDA (Food and Drug Administration) [13]. Additionally, studies addressing the milk microbiome have revealed several members of this group as non-pathogenic commensals [15]. Furthermore, studies by Crispie et al. [16], Klostermann et al. [10] and Beecher et al. [17] have shown promising results after administering live lactic acid bacteria cultures to the bovine udder.

According to Frola et al. [12] the formation of a beneficial biofilm by colonising the inner surfaces of the udder and thereby building a barrier against pathogenic microorganisms is an important factor for the probiotic potential of lactic acid bacteria. Furthermore, the adhesion capacity to the epithelium accounts for the ability of bacterial strains to sustain their antimicrobial effects over time by maintaining their presence in the host [15],[18],[19]. These effects can be achieved by the secretion of inhibitory substances such as bacteriocins and other antimicrobial compounds [19]. Interaction with the epithelium might also trigger immune responses in the lamina propria this being commonly regarded as an important benefit exerted by probiotics [14]. The close association between epithelium and bacteria which is necessary for the probiotic features mentioned above is established in two steps: In the first step, unspecific binding through physicochemical interaction occurs. Subsequently, receptor-ligand interactions mediated by specific adhesins lead to irreversible bacterial attachment [20].

The aim of the present study was to examine the biofilm formation and adhesion capacity of 13 LAB strains to evaluate their probiotic potential. All of the strains inhibited the growth of at least one mastitis-causing pathogen *in vitro* [21]. Since the exact surface properties of the udder's glandular epithelium and the surrounding conditions are currently unknown, differently charged surfaces were offered to the bacteria in order to gain information about their behaviour under a broader spectrum of conditions.

## 2. Materials and methods

### 2.1. Microorganisms

In order to assess biofilm formation and adhesion capacity of LAB, 13 strains (Table 1) were selected from the strain collection of Faculty II, Department for Bioprocess Engineering and Microbiology of the University of Applied Sciences and Arts Hannover, Germany. All of the strains had shown ability to inhibit the growth of certain mastitis-causing pathogens in previous experiments [21]. The strains were stored in MRS broth with 20% glycerin at −80 °C. Identification of the strains was performed with a commercial Analytical Profile Index assay (API 50 CH with API 50 CHL Medium, Biomérieux, Nürtingen, Germany) according to the manufacturer's instructions [21]. For the strains *Lactobacillus* (*Lb*.) *paracasei* subsp. *paracasei* 78/37 and *Lb. plantarum* 118/37, Diepers et al. [21] had previously confirmed the API results by 16S rDNA gene amplification. Nine strains (*Lb*. *plantarum* 2/37, *Lactococcus* (*Lc*.) *lactis* subsp. *lactis* 33/30, *Lb. paracasei* subsp. *paracasei* 35/37, *Lb. paracasei* subsp. *paracasei* 42/37, *Lb. brevis* 46/30, *Lb. paracasei* subsp. *paracasei* 78/37, *Lb. brevis* 104/37, *Lb. plantarum* 118/37, *Lb. paracasei* subsp. *paracasei* 123/37) were isolated from quarter milk samples with normal secretion (no pathogen detection in foremilk samples, somatic cell count < 100,000/mL). One strain was isolated from a bulk milk sample (*Lb. buchneri* SX.A.2) and one more from a bedding sample (*Lb. plantarum* 6E). Additionally, the reference strains *Lc. lactis* subsp. *lactis* ATCC 11454 and *Lb. rhamnosus* ATCC 7469 were included in the study.

**Table 1.** Strains used in the study.

Strain	Origin
*Lb. rhamnosus* ATCC 7469	American Type Culture Collection (ATCC)
*Lc. lactis* subsp. *lactis* ATCC 11454	
*Lb. plantarum* 2/37	Quarter milk samples
*Lc. lactis* subsp. *lactis* 33/30	(No pathogen detected, somatic cell count < 100.000/mL)
*Lb. paracasei* subsp. *paracasei* 35/37	
*Lb. paracasei* subsp. *paracasei* 42/37	
*Lb. brevis* 46/30	
*Lb. paracasei* subsp. *paracasei* 78/37	
*Lb. brevis* 104/37	
*Lb. plantarum* 118/37	
*Lb. paracasei* subsp. *paracasei* 123/37	
*Lb. buchneri* SX.A.2	Bulk milk sample
*Lb. plantarum* 6E	Bedding sample

### 2.2. Assessing of biofilm formation

For the biofilm assay bacteria were grown in MRS broth described by Leccese Terraf et al. [22], either containing 0.1% Tween 80 (PanReac AppliChem, Barcelona, Spain) (v/v) or not, since Leccese Terraf et al. [22] found this component to influence biofilm formation in some LAB. For this reason, we decided to include broth with and without Tween 80 in the study. The broth was filled into glass tubes and into polypropylene tubes (Greiner Bio-One GmbH, Frickenhausen, Germany). To investigate the ability of the bacteria to form biofilms, an assay previously described by Leccese Terraf et al. [22] was used with certain modifications; a photometer loaded with polymethylmethylacrylate-(PMMA)-cuvettes (Brand, Wertheim, Germany) was used instead of a microplate reader to measure the optical density (OD) of the samples.

Prior to this investigation, the strains had been transferred from the frozen stock culture to MRS broth containing Tween 80 and incubated aerobically for 24 h at 37 °C. Subsequently, a second subculture had been made and incubated under the same conditions. The third subculture had been performed in duplicate using complete as well as Tween 80-depleted MRS broth.

An aliquot of 1 mL from the third subculture was centrifuged for 15 min, at 6000 × g. After decanting the supernatant, the pellet was resuspended in 1 mL 0.85% NaCl (w/v) solution. The suspension's optical density was then adjusted to 0.6 at 540 nm wavelength [22] corresponding to 7 log_10_ CFU (colony-forming units)/mL for each strain by adding 0.85% NaCl (w/v) solution. From this suspension an inoculum of 200 µl was transferred to 5 mL MRS broth with or without Tween 80, resembling the composition of the subculture's medium from which the inoculum had been created. Both kinds of broth, Tween 80-depleted and complete, had been filled in glass and in polypropylene tubes resulting in four combinations of medium and surface that were offered to each strain. Glass and polypropylene were chosen because of their different surface charge. The latter is hydrophilic for glass and hydrophobic for polypropylene. The inoculated broths were incubated aerobically at 37 °C. After 72 h the broth in the tubes was discarded and the formed biofilms were first washed with PBS [22] and then stained with 5 mL of an aqueous 0.1% crystal violet (Merck KGaA, Darmstadt, Germany) (w/v) solution for 30 min. A glass and polypropylene tube each without inoculated broth were included into the staining procedure to serve as control. Crystal violet was then discarded and unbound dye washed away with tap water before extracting crystal violet from the biofilm with 5 mL of 96% ethanol. To quantify the biomass of this solution the optical density was measured at 570 nm wavelength with a photometer. The control corresponding to the tube's material served as blank value. The experiment was performed in triplicate.

### 2.3. Adhesion capacity

In order to assess the strains' adhesion capacity to the udder epithelium the strains were subjected to a trial which was performed according to Frola et al. [12] and Otero and Nader-Macías [23]. The experimental design was modified by implementing some aspects that were taken from the studies of Greene and Klaenhammer [24] and Roos and Jonsson [25], mainly referring to the pre-treatment of the bacterial strains (see below). Further modifications were performed by replacing Eppendorf tubes for the pre-incubation with 96-well-microplates (Greiner Bio-One GmbH, Frickenhausen, Germany). Additionally, glandular epithelial cells were used.

Bacteria were grown in complete MRS-broth. Three consecutive subcultures were made before the assay. Each of them was incubated for 24 h at 37 °C.

The epithelial cell suspension was created by scraping off cells from the lactiferous sinus and the large ducts of a fresh bovine udder. The udder had been obtained from a slaughterhouse in North-Rhine-Westphalia, Germany and transported to the laboratory in Hannover, while being kept under refrigerated conditions (8 °C). Prior to the experiment the udder had been washed thoroughly with tap water. The cells were then suspended in 1 mL of Eagle Minimum Essential Media (MEM) (Thermo Fisher Scientific, Waltham, MA USA) and washed three times in 1 mL MEM. Exemption from bacterial contaminations was ensured by Gram-staining followed by examination under a light microscope. Furthermore, the cell viability was determined by the Trypanblue Exclusion Method (Thermo Fisher Scientific, Waltham, MA USA). The cell concentration was adjusted to 10^5^/mL viable cells and the suspension was stored at 8 °C until use.

The bacterial cell suspension for the trial was prepared by adjusting the optical density of the third subculture to 0.6 at 540 nm wavelength as previously described for the biofilm assay. Instead of 0.85% NaCl solution, PBS was used in this assay. To determine the chemical nature of the adhesion factors involved, different treatments were applied decreasing the adhesion parameters due to enzymatic degradation of proteins or lipids or oxidization of carbohydrate structures by sodium meta-periodate. We assumed that decreased adhesion after pre-treatment with an enzymatic or a chemical agent accounted for the relevant adhesin to belong to the specific class of macromolecule disintegrated by the agent. For each strain, six suspensions, each having a volume of 0.3 mL were prepared. The LAB were then pre-incubated with MEM, PBS, 5 mg/mL trypsin (14584 U/mg, Sigma Alldrich Chemie GmbH, Munich, Germany), 100 µg/mL proteinase K (35.1 mAnsonU/mg, PanReac AppliChem, Barcelona, Spain), 0.05 mmol/mL sodium meta-periodate (PanReac AppliChem, Barcelona, Spain) and 2 mg/mL lipase (100–500 U/mg in olive oil, Sigma Alldrich Chemie GmbH, Munich, Germany) by adding 0.3 mL of the latter solutions to the bacterial suspensions. All chemicals and enzymes were dissolved in PBS, included in the assay as a pre-treatment chemical to serve as control. As previously mentioned, the epithelial cell suspension was prepared in MEM. This served as negative control. Bacterial suspension and the chemical or enzymatical solutions were incubated together for 1 h at 37 °C according to Greene and Klaenhammer [24]. The bacteria were then washed twice in MEM.

The actual adhesion assay was performed by adding 100 µl of the pre-incubated bacterial cell suspension to 100 µl of the epithelial cell suspension in a polypropylene 96-well microplate (Greiner Bio-One GmbH, Frickenhausen, Germany). Three wells on each microplate were filled with 200 µl pure MEM and served as control. Incubation time was set again to 1 h at 37 °C while keeping the plates under gentle agitation [23]. After washing the epithelial cells and bacteria in MEM three times, gram-staining of the mixed suspension was performed and the results evaluated using light microscopy. As described by Otero and Nader-Macías [23], the percentage of adhesion and adhesion index were assessed by counting ten randomised fields of vision.

The percentage of adhesion was defined as follows:

$$\frac{\mathrm{Number}\mathrm{of}\mathrm{epithelial}\mathrm{cells}\mathrm{with}\mathrm{adherent}\mathrm{bacteria}}{\mathrm{Total}\mathrm{number}\mathrm{of}\mathrm{epithelial}\mathrm{cells}}\times 100$$ (1)

The adhesion index was defined as follows:

$$\frac{\mathrm{Number}\mathrm{of}\mathrm{bacteria}\mathrm{adhering}\mathrm{to}\mathrm{epithelial}\mathrm{cells}}{\mathrm{Number}\mathrm{of}\mathrm{epithelial}\mathrm{cells}\mathrm{with}\mathrm{adherent}\mathrm{bacteria}}$$ (2)

The assay was performed in triplicate.

### 2.4. Statistical analysis

The statistical analysis of the data obtained was performed with IBM SPSS Statistics 23. For each assay performed, the mean values of all the three repetitions were calculated. The data revealed normal distribution which was confirmed by the Kolmogorov-Smirnov-Test. In order to compare the strains' behaviour concerning biofilm formation and adhesion under the different experimental conditions, the results of both assays were examined using Pearson's-correlation-test. The comparison of the mean values was performed using an analysis of variance (ANOVA).

## 3. Results

### 3.1. Biofilm formation

**Figure 1.**
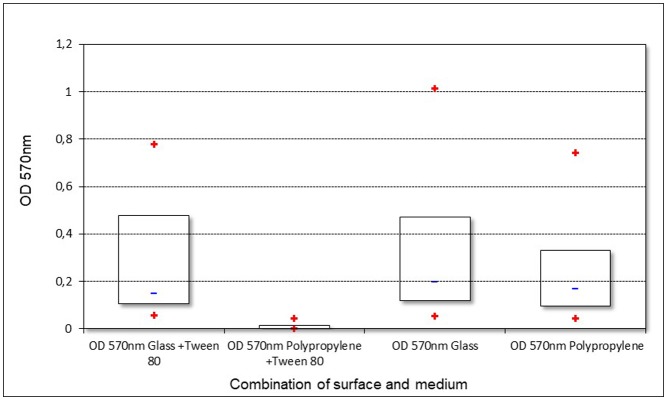
Biofilm formation of all strains with different combinations of surface and medium (+ means min/max; − means median).

**Figure 2.**
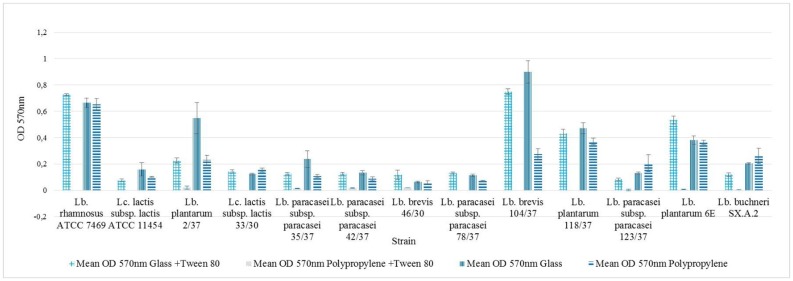
Optical density as indicator for biofilm formation, results expressed as arithmetical mean ± standard error of the mean.

The strains' ability to form a biofilm was examined by an assay which combined two different media and two differently charged surfaces, each strain therefore being subjected to four trials. The results are shown in Figure 1 ordered by combination of surface and media. In Figure 2 the results for each single strain are given. All of the strains revealed the ability of biofilm formation, nevertheless the OD values in each trial varied among the strains. Furthermore, the assay showed varying results for the same strain on different combinations of surface and medium. The overall lowest values were obtained when combining a hydrophobic polypropylene surface and MRS broth containing Tween 80.

*Lb. brevis* 104/37 revealed the highest OD values during the experiment, with an arithmetical mean of 0.9 ± 0.08 (standard error of the mean) growing in MRS broth without Tween 80 on a glass surface (see Figure 2). The lowest OD values were measured for *Lb. rhamnosus* ATCC 7469, *Lc. lactis* subsp. *lactis* ATCC 11454, *Lc. lactis* subsp. *lactis* 33/30, *Lb. paracasei* subsp. *paracasei* 78/37, *Lb. brevis* 104/37 and *Lb. plantarum* 118/37, all being unable to build a detectable biofilm on polypropylene when grown in MRS broth containing Tween 80. The mean OD (570 nm) value for the assay was 0.21 ± 0.03. For five of the 13 strains OD values higher than the mean were found in three of the trials (OD 570 nm glass + Tween 80, OD 570 nm glass, OD 570 nm polypropylene) namely *Lb. rhamnosus* ATCC 7469, *Lc. lactis* subsp. *lactis* ATCC 11454, *Lb. plantarum* 2/37, *Lb. brevis* 104/37, *Lb. plantarum* 118/37 and *Lb. plantarum* 6E.

Correlations between the results of the assay according to Pearson were found as described in Table 2.

**Table 2.** Pearson-correlations between biofilm trials with different combinations of surface and medium (*p* < 0.05).

r = Coefficient of correlation	Biofilm on glass (Tween 80)	Biofilm on polypropylene
Biofilm on glass	0.89	0.68
Biofilm on polypropylene	0.80	

The ANOVA revealed significant differences between the mean values of the four trials and between most of the strains (*p* < 0.05).

### 3.2. Adhesion capacity

**Figure 3.**
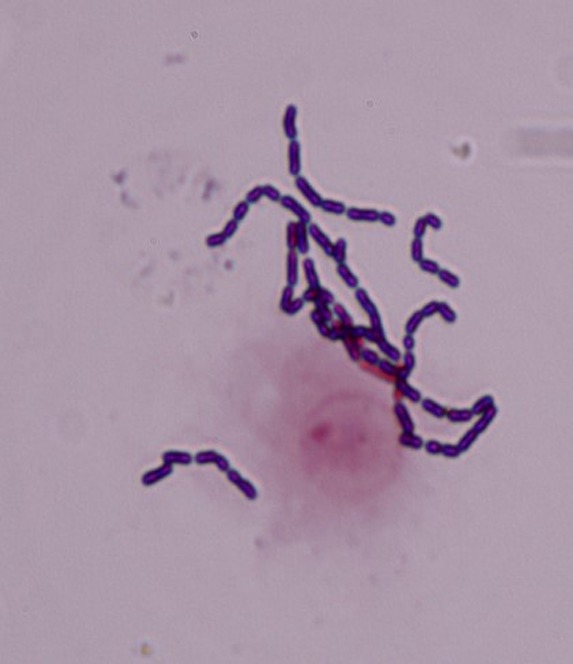
Gram stained *Lb.* rhamnosus ATCC 7469 adhering to glandular epithelial cell of bovine udder after pre-incubation in MEM.

**Figure 4.**
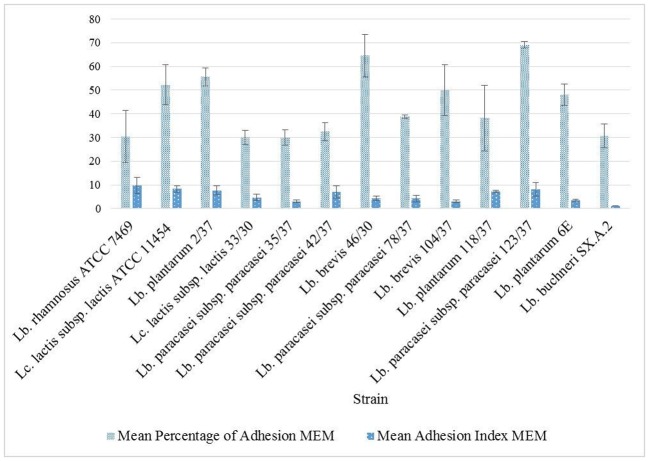
Adhesion capacity after pre-incubation in MEM, results expressed as arithmetical mean ± standard error of the mean.

To assess the adhesion capacity to glandular epithelial cells of the udder, bacterial cells previously pre-incubated in MEM were incubated together with freshly gained epithelial cells in MEM and evaluated under a light microscope (Figure 3). The ability to adhere to glandular epithelial cells from the bovine udder was present among all of the strains tested during this study. However, the degree of adhesion varied between the different strains (Figure 4). The mean percentage of adhesion after incubating cells and bacteria in MEM was approximately 44% ± 3.8 with a mean adhesion index of approximately 5.5 ± 0.7 bacteria. *Lb. paracasei* subsp. *paracasei* 123/37 displayed the highest percentage of adhesion with about 70% ± 1.4 throughout all three repetitions of the assay. *Lb. brevis* 46/30's average percentage of adhesion was approximately 65% ± 9 and *Lc. lactis* subsp. *lactis* ATCC 11454, *Lb. plantarum* 2/37, *Lb. brevis* 104/37 and *Lb. plantarum* 6E revealed values of approximately 50%. The other strains showed a lower percentage of adhesion, the lowest values being approximately 30% of the cells with bacteria adherent. There was no significant correlation found between the percentage of adhesion and adhesion index in MEM.

### 3.3. Chemical nature of adhesion factors

**Figure 5.**
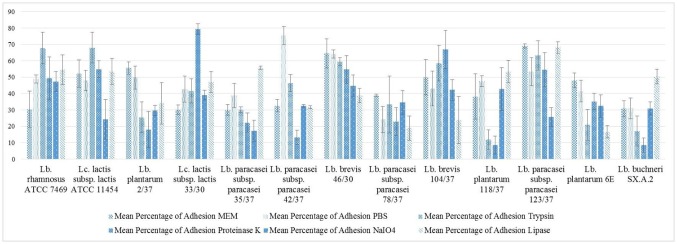
Percentage of adhesion, results expressed as arithmetical mean ± standard error of the mean.

**Figure 6.**
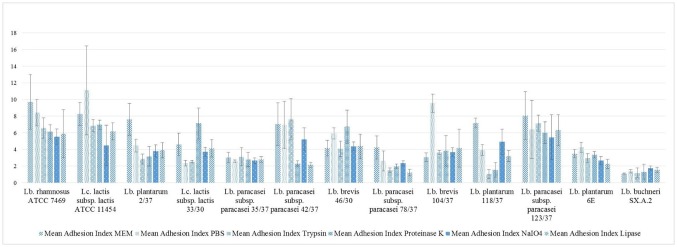
Adhesion index, results expressed as arithmetical mean ± standard error of the mean.

The results for the single strains are shown in Figure 5 and Figure 6. After pre-treatment with PBS, in some cases an aberration of the percentage of adhesion from the results obtained after pre-treatment with MEM for the same strains was observed. For *Lb. rhamnosus* ATCC 7469 and *Lb. paracasei* subsp. *paracasei* 42/37, adhesion compared to the results obtained after MEM pre-treatment increased after pre-incubation in PBS from approximately 30% cells with bacteria adherent to almost 49% for *Lb. rhamnosus* ATCC 7469 and from 32% to 75% for *Lb. paracasei* subsp. *paracasei* 42/37.

In some strains the chemical and enzymatical pre-treatment resulted in changes in the percentage of adhesion compared to the values recorded after PBS and MEM treatment. *Lc. lactis* subsp. *lactis* ATCC 11454 and *Lb. paracasei* subsp. *paracasei* 123/37 showed a decreased percentage of adhesion after treatment with sodium meta-periodate. In *Lb. plantarum* 118/37 and *Lb. buchneri* SX.A.2 similar observations were made after pre-treating the bacterial cells with trypsin and proteinase K, whereas the percentage of adhesion in *Lb. paracasei* subsp. *paracasei* 42/37 was only reduced by proteinase K pre-treatment. Furthermore, proteinase K and sodium meta-periodate both caused a considerable reduction in the adhesion capacity of *Lb. paracasei* subsp. *paracasei* 35/37. *Lb. plantarum* 6E revealed a diminished ability to adhere to epithelial cells of the bovine udder after being subjected to trypsin and to lipase treatment. In *Lb. brevis* 104/37 only lipase treatment caused a decrease in the percentage of adhesion. All of the four pre-treatments led to a reduction in the percentage of adhesion in *Lb. plantarum* 2/37 and *Lb. brevis* 46/30. In contrast to these findings apparently neither of the enzymes, nor sodium meta-periodate were capable of reducing the percentage of adhesion in *Lb. paracasei* subsp. *paracasei* 78/37 and *Lc. lactis* subsp. *lactis* 33/30.

In some of the strains, increased adhesion was detected after protease treatment; namely *Lb. rhamnosus* ATCC 7469, *Lc. lactis* subsp. *lactis* ATCC 11454, *Lc. lactis* subsp. *lactis* 33/30 and *Lb. brevis* 104/37 which adhered to a higher extent to the mammalian cells after pre-incubation with trypsin and/or proteinase K.

Correlations according to Pearson were found for the percentage of adhesion after proteinase K and trypsin-pre-treatment (r = 0.71, *p* < 0.05), between the percentages of adhesion after sodium meta-periodate treatment and the results of the biofilm assay employing glass and complete MRS broth (r = 0.59, *p* < 0.05) and between the percentage of adhesion after pre-incubation with PBS and OD values of biofilms grown on polypropylene in complete MRS broth (r = 0.57, *p* < 0.05). Correlations between the percentage of adhesion values and adhesion indices after protease pre-treatment are shown in Table 3.

**Table 3.** Pearson-correlations between adhesion parameters after protease pre-treatment (* = *p* < 0.05).

r = Coefficient of correlation	% Adhesion (trypsin)	% Adhesion (proteinase K)	Adhesion Index (trypsin)
Adhesion Index (proteinase K)	0.78*	0.87*	0.53*
Adhesion Index (trypsin)	0.8*	0.33	

Significant differences in the mean percentage of adhesion occurred between the trial using sodium meta-periodate as pre-treatment chemical and the trials using MEM, PBS, trypsin and lipase as well as between the trial using proteinase K and the trials using MEM and PBS (*p* < 0.05). The ANOVA revealed no significant differences between the mean adhesion index after PBS and MEM pre-treatment (*p* > 0.05). However, the mean adhesion indices of these two trials were significantly different from the mean adhesion indices after pre-treatment with the three enzymes and sodium meta-periodate (*p* < 0.05).

## 4. Discussion

The present study dealt with two assays, investigating the ability of thirteen LAB strains to form a biofilm and to adhere to glandular epithelial cells of the bovine udder *in vitro*. The aim of the study was to assess the strains' probiotic potential. Knowledge about the specific surface conditions and the bacterial properties influencing adhesion and subsequent biofilm formation on the udder's epithelium is still insufficient [15]. Some authors regard bacterial hydrophobicity as crucial for adhesion [26], others found no correlation between this trait and the adhesive properties of bacteria [27]. According to An and Friedman [6], bacteria favour a substrate for adhesion resembling their own surface charge. For these reasons we included both a hydrophobic and a hydrophilic surface in our study.

All of the examined strains were able to form a biofilm and to adhere to epithelial cells; however, not to the same extent. Formation of biofilm was apparently more pronounced on hydrophilic than on hydrophobic surfaces (Figure 1). This is in line with the previously mentioned studies of An and Friedman [6], since glass as well as bacterial surfaces are normally negatively charged or at least hydrophilic. Additionally, Tween 80 appeared to cause considerably decreased adhesion, especially to hydrophobic polypropylene, leading to a diminished or in some strains even absent biofilm formation. These results are inconsistent with the study findings of Donlan [5] who stated that bacterial adherence was more distinct on hydrophobic surfaces. However, our results are in line with the findings of Leccese Terraf et al. [22] who observed that biofilm formation by LAB on polystyrene, which has a hydrophobic surface, occurred only in MRS broth without Tween 80, while this detergent did not inhibit biofilm formation in MRS broth on glass. We did not investigate possible alterations of the surface and its charge by a conditioning film which could form on the surface after exposure to the medium. Interaction with the conditioning film could possibly explain the inhibition of biofilm formation by Tween 80. The significance of surface conditioning for bacterial adhesion to a surface as well as the antagonistic effect of certain detergents on biofilm formation were already described by ZoBell in 1943 [28]. Significant positive correlations were found between the two trials using glass and the trial using polypropylene and MRS broth in which Tween 80 was omitted. However, no correlation between the trial on polypropylene in complete broth and any of the other three trials could be observed. These findings suggest that biofilm formation was more dependent on strain and medium than on the surface charge in this assay. This is in line with the results of Hagi et al. [15] who also found adhesion to be highly strain-dependent during their research.

In contrast to previous *in vitro* studies using epithelial cells from the teat canal of a bovine udder [12], we took cells from the udder's glandular tissue in order to gain information about LABs' ability to colonise the proximal regions of the udder where pathogens might settle. The adhesion capacity was assessed by two parameters; namely, the percentage of adhesion and the adhesion index. Between these two parameters no correlation was found. This implies that a higher number of cells with adherent bacteria did not necessarily lead to a higher number of bacteria attached to the single cells.

Discrepancies in adhesion parameters after pre-treatment with PBS and medium were previously reported by Li et al. [29] who observed a decreased adhesion index after resuspending bacterial cells in in fresh MRS broth compared to the adhesion index in PBS. They stated that these results could point to steric hindrance by integrated chemicals from the broth preventing bacterial adhesion. In our study we found the percentage of adhesion to be clearly increased in *Lb. rhamnosus* ATCC 7469 and *Lb. paracasei* subsp. *paracasei* 42/37 after pre-incubation in PBS compared to the results obtained in MEM. The other strains displayed more similar values for the mean percentage of adhesion in PBS and MEM and the ANOVA revealed no significant differences between these two solutions for the whole assay. Additionally, the mean adhesion indices after pre-treatment with MEM and PBS were not significantly different from each other, but there was a difference from the mean adhesion indices after chemical or enzymatical pre-treatment. The effect of MEM therefore seems to be strain-dependent and might be due to steric hindrance.

Chemical pre-treatment with sodium meta-periodate revealed carbohydrate molecules on the bacterial surface to be probably involved in adhesion to the glandular epithelial cells of the bovine udder by *Lc. lactis* subsp. *lactis* ATCC 11454 and *Lb. paracasei* subsp. *paracasei* 123/37. In *Lb. plantarum* 118/37, *Lb. buchneri* SX.A.2 and *Lb. paracasei* subsp. *paracasei* 42/37, adhesion was probably mediated by proteins. Yet, the structure of the proteins might differ since the adhesion of *Lb. paracasei* subsp. *paracasei* 42/37 was only reduced after pre-treatment with proteinase K and not after pre-treatment with trypsin, whereas both enzymes achieved a reduced percentage of adhesion in the other two strains. Still, the values for the percentage of adhesion after pre-treatment with the two proteases used in the study correlated with each other as well as with the adhesion indices after protease treatment, indicating that in most of the cases they had a similar effect on the same strain. For some of the strains a decreased adhesion was achieved with more than one enzyme class or chemical, implying that different classes of macromolecules were involved in adhesion to glandular tissue. In their studies Greene and Klaenhammer [24] also found adhesion to be mediated by more than one mechanism. For *Lb. paracasei* subsp. *paracasei* 35/37 it can be assumed that carbohydrates as well as proteins or glycoproteins on the bacterial surface might play a dominating role concerning adherence to epithelial cells. The adhesive properties of *Lb. plantarum* 6E were probably mediated by proteins and lipids since adhesion was influenced by trypsin and lipase. During the trials for *Lb. plantarum* 2/37 and *Lb. brevis* 46/30 all of the three classes of adhesins appeared to be involved, whereas none of the investigated macromolecules seemed to be responsible for the adhesion of *Lb. paracasei* subsp. *paracasei* 78/37 and *Lc. lactis* subsp. *lactis* 33/30. Increased adhesion following protease treatment was observed in *Lb. rhamnosus* ATCC 7469, *Lc. lactis* subsp. *lactis* ATCC 11454, *Lc. lactis* subsp. *lactis* 33/30 and *Lb. brevis* 104/37. This phenomenon was previously described by Otero and Nader-Macías [23] who also observed an increase in adhesion parameters after applying proteinase K and/or trypsin to their LAB strains.

The crystal violet assay is a common method for quantifying biofilms. It is widely used as it is easy to perform and allows a high throughput of samples. Furthermore, crystal violet binds non-specifically to the bacterial cells as well as to matrix components, therefore allowing quantification of the total biofilm mass [30]. The tube method can also be performed to determine biofilm formation, staining bacterial cells with crystal violet; however, it only allows qualitative or semi-quantitative biofilm assessment. Investigating biofilm formation by growing bacteria on congo red agar followed by evaluation of the colony morphology is a further qualitative approach [9]. Another frequently used quantitative method involves scraping off biofilms from their surface, resuspending them and determining the number of colony-forming units (CFU) by cultivation. However, Fernández Ramírez et al. [30] found poor correlation between the culture-based approach and the crystal violet assay probably because mature biofilms contain only few culturable cells embedded in a thick layer of matrix components which cannot be detected by culture-based methods.

Adhesion to epithelial cells of the udder's glandular tissue was investigated in this study by applying a method using freshly gained cells which were incubated together with pre-treated bacteria. This method is often used in probiotic research not only concerning the bovine udder [23]. Alternative approaches used cultured bovine mammary gland epithelial cells which were detached from their surface after the adhesion assay the colony-forming units being determined among the adherent bacteria thereafter [15]. However, Opdebeeck et al. [31] suspected cultured epithelial cells to lose surface receptors crucial to bacterial adhesion during differentiation.

Searching for adhesins Kinoshita et al. [32] proposed a genotypic screening method by analysing moonlighting proteins of LAB mediating adhesion to porcine intestinal mucus. To our knowledge, no adhesins mediating LAB's adhesion to epithelial cells of the mammary gland have been identified in the genetic level, yet. The results obtained in this study represent an approach to identify the macromolecules involved into LAB's adhesion to epithelial cells by characterising some of their basic chemical properties. Still, further research is required to identify adhesins in order to enhance knowledge about potentially probiotic bacterial interaction with the host's epithelium [15]. Our trials revealed *Lb. rhamnosus* ATCC 7469, *Lb. plantarum* 2/37, *Lb. brevis* 104/37, *Lb. plantarum* 118/37, *Lb. plantarum* 6E, *Lb. brevis* 46/30 and *Lb. paracasei* subsp. *paracasei* 123/37 to have the most pronounced biofilm forming ability and/or adhesion capacity. They are therefore promising candidates for further research concerning their impact on pathogenic biofilms as well as for *in vivo* experiments. Interestingly, the values of the biofilm trial combining complete MRS broth and a hydrophobic polypropylene surface alone showed significant positive correlation with the percentage of adhesion after pre-incubation in PBS. Possibly, the polypropylene trial in complete broth is more suitable for predicting *in vitro* adhesion to epithelial cells than the other biofilm trials (glass + Tween 80, glass, polypropylene + Tween 80). In a milky environment, conditioning films composed of fat and protein molecules can occur and cause alterations in the surface charge [33],[34]. Hamadi et al. [33] found an inhibitory effect of a fatty conditioning film on bacterial adhesion. Therefore, the ability of binding to a hydrophobic substrate might be an advantage under *in vivo* conditions. The fact that in some of the strains adhesion to polypropylene still occurred in the presence of Tween 80 could be due to another mechanism for binding hydrophobic substrates, such as non-specific binding related to surface hydrophobicity [26]. However, knowledge about the *in vivo* conditions for colonisation of the udder is still insufficient and deserves further investigation. Additionally, the question of safety of the strains regarding the host as well as consumers of milk products needs to be answered. Even though the strains investigated were supposedly harmless commensals, mostly isolated from healthy udders, there is evidence of mastitis in cattle [35] and infections in humans [13] caused by LAB. These issues still need to be covered by future research including *in vivo* experiments in order to achieve a probiotic alternative for prophylaxis or treatment of bovine mastitis and to improve our understanding of interactions between host, pathogens and probiotic LAB.

## 5. Conclusions

The present study focussed on evaluating the probiotic potential of LAB by investigating their ability to form a biofilm on glass and polypropylene as well as their adhesion capacity to mammary glandular cells *in vitro*. The results recommend seven of the investigated 13 strains (*Lc. lactis* subsp. *lactis* ATCC 11454, *Lb. rhamnosus* ATCC 7469, *Lb. plantarum* 2/37, *Lactococcus lactis* subsp. *lactis* 33/30, *Lb. paracasei* subsp. *paracasei* 35/37, *Lb. paracasei* subsp. *paracasei* 42/37, *Lb. brevis* 46/30, *Lb. paracasei* subsp. *paracasei* 78/37, *Lb. brevis* 104/37, *Lb. plantarum* 118/37, *Lb. paracasei* subsp. *paracasei* 123/37, *Lb. buchneri* SX.A.2, *Lb. plantarum* 6E) for further trials assessing their safety for consumers and patients as well as their efficacy in colonising the bovine udder in order to treat or prevent infection. Further research involving *in vivo* experiments is necessary to overcome this knowledge gap.
